# Therapeutic Potential of Leaves from *Fridericia chica* (Bonpl.) L. G. Lohmann: Botanical Aspects, Phytochemical and Biological, Anti-Inflammatory, Antioxidant and Healing Action

**DOI:** 10.3390/biom12091208

**Published:** 2022-08-31

**Authors:** Adriane Dâmares de Sousa Jorge Batalha, Damy Caroline de Melo Souza, Rosmery Duran Ubiera, Francisco Celio Maia Chaves, Wuelton Marcelo Monteiro, Felipe Moura Araújo da Silva, Hector Henrique Ferreira Koolen, Antônio Luiz Boechat, Marco Aurélio Sartim

**Affiliations:** 1 Basic and Applied Graduate Program—PPGIBA, Biological Science Institute, Federal University of Amazonas, Manaus 69080-900, Brazil; 2 Embrapa Western Amazonia, Manaus 69010-970, Brazil; 3 Tropical Medicine Graduate Program, Amazonas State University—UEA, Manaus 69040-000, Brazil; 4 Tropical Medicine Foundation Heitor Vieira Dourado (FMT-HVD), Manaus 69040-000, Brazil; 5 Multidisciplinary Support Center, Federal University of Amazonas—UEA, Manaus 69080-900, Brazil; 6 Research Group in Metabolomics and Mass Spectrometry, Amazonas State University, Manaus 690065-130, Brazil; 7 Laboratory of Innovative Therapies, Department of Parasitology, Amazonas State University—UEA, Manaus 69080-900, Brazil; 8 Research & Development Department, Nilton Lins Foundation, Manaus 69058-030, Brazil

**Keywords:** *Fridericia chica*, phytochemistry, anti-inflammatory, antioxidant, wound healing

## Abstract

Plants of the species *Fridericia chica* (Bonpl.) L. G. Lohmann (Bignoniaceae), which are widely distributed in Brazil and named crajiru in the state of Amazonas, are known in folk medicine as a traditional medicine in the form of a tea for the treatment of intestinal colic, diarrhea, and anemia, among other diseases. The chemical analysis of extracts of the leaves has identified phenolic compounds, a class of secondary metabolites that provide defense for plants and benefits to the health of humans. Several studies have shown the therapeutic efficacy of *F. chica* extracts, with antitumor, antiviral, wound healing, anti-inflammatory, and antioxidant activities being among the therapeutic applications already proven. The healing action of *F. chica* leaf extract has been demonstrated in several experimental models, and shows the ability to favor the proliferation of fibroblasts, which is essential for tissue repair. The anti-inflammatory activity of F. chica has been clearly demonstrated by several authors, who suggest that it is related to the presence of 3-deoxyanthocyanidins, which is capable of inhibiting pro-inflammatory pathways such as the kappa B (NF-kB) nuclear transcription factor pathway. Another important effect attributed to this species is the antioxidant effect, attributed to phenolic compounds interrupting chain reactions caused by free radicals and donating hydrogen atoms or electrons. In conclusion, the species *Fridericia chica* has great therapeutic potential, which is detailed in this paper with the objective of encouraging new research and promoting the sum of efforts for the inclusion of herbal medicines in health systems around the world.

## 1. Introduction

**Figure 1:**
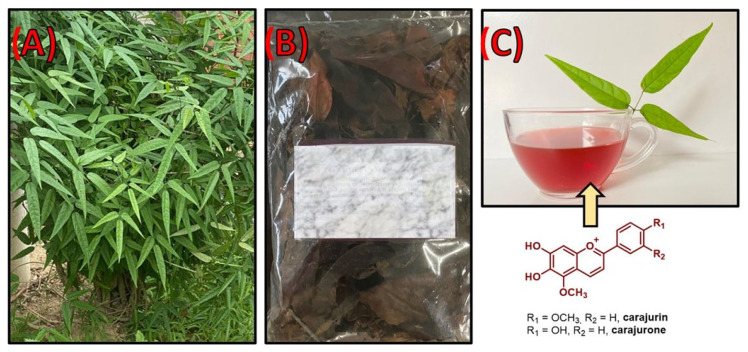
*Fridericia chica* (Bonpl.) L. G. Lohmann: specimen and presentations forms. (**A**) Arboreal specimen of adult *F. chica in situ* in the city of Manaus, Amazonas Brazil. (**B**) Dried leaves of *F. chica* as marketed in the city of Manaus, Brazil. (**C**) Tea obtained from the dried leaves of *F. chica* and its main compounds responsible for the characteristic color. Photos: Author.

*Fridericia chica* (Bonpl.) L. G. Lohmann (Figure 1) is a liana or creeper that belongs to the family Bignoniaceae, which comprises about 120 genera and 800 species [1]. The largest number of species of this family is found mainly in tropical and subtropical regions, with Brazil and the African continent as the two major centers of geographical distribution. In Brazil, the species of the family Bignoniaceae do not have a unique habitat and are widely distributed in the region of the legal Amazon, as well as throughout the southern region of the country as far down as Rio Grande do Sul. In addition, plants of this family can be found in the Cerrado and Atlantic Forest biomes [2,3,4]. *F. chica* was first described by Cronquist [5] as *Arrabidaea chica*, belonging to the division Magnoliophyta, class Magnoliopsida, subclass Asteridae, order Scrophulariales, family bignoniaceae, and genus *Arrabidaea*. However, its classification has been altered due to recent taxonomic changes, such as the inclusion of several species of the genus *Arrabidaea* in the genus *Fridericia*. This change has affected the classification of its varieties *A. chica var. acutifolia* (DC.) Bureau, *A. chica var. angustifolia* Bureau & K. Schum, *A. chica var*. *cuprea* Bureau & K. Schum, *A. chica var. thyrsoidea* (DC.) Bureau and *A. chica var. viscida* Donn.Sm [6].

Botanically, *Fridericia chica* are described as woody, shrub-like, or arboreal plants, as well as climbing plants, with that leaves measure between 18–20 cm in length when mature. They have opposite crossed leaves, compound bi- or trifoliate, however, with the terminal foliole modified in tendrils in the elevated part of the branches. The folioles have an oblong-lanceolate shape, cartaceous, with an obtuse base, acute apex and herbaceous consistency. The surface of the folioles is smooth, and the rib is of the peninerveal type, i.e., the secondary ribs branch from the main rib [3,7,8,9]. The flowers are campanulate, resemble the shape of a bell, and have pink or violet coloration [10,11]. 

*F. chica* occurs in Central American countries, the Caribbean, and mainly in South American countries, such as Guyana and French Guiana, and especially in Brazil. In the latter it has wide geographical distribution, with confirmed occurrence in all regions and phytogeographic domains, including the Amazon, the Caatinga, the Cerrado, the Atlantic Forest, and the Pantanal biomes. With regard to the type of vegetation, *F. chica* can be found in floodplains or forests of various types, such as riparian, flooded forests, and terra firma, among others [6].

## 2. Revision Strategy 

An extensive literature review was carried out using different scientific electronic sources, including databases such as Scifinder, Pubmed, Scopus, Web of Science, and Google Scholar. The study databases included original papers published in peer reviewed journals, books, dissertations, and theses, and all data of scientific interest written or translated into English published prior to July 2022 was considered. The keywords “Arrabidae chica” and “Fridericia chica” alone and in combination with änflammation”, inflammatory”, änti-inflammatory”, “oxidative stress”, “antioxidant”, “wound healing”, and “healing”. In addition, the names of all phytochemical compounds were used in the search. 

## 3. Ethnobotany

In Brazil, *F. chica* is popularly known as cajuru, carajiru, carajunu, carajuru, chica, china, cipó-cruz, cipó-pau, coá-piranga, crajiru, crajuru, cuica, guajuru, guajuru-piranga, guarajuru, oajuru, oajuru-piranga, paripari, pariri, and piranga, among others [2,6,10]. Its mature leaves, collected from plants ranging from 50 cm to 2 m, are used in the form of tea (dried form), which has an astringent function and can be used against intestinal colic, diarrhea, anemia, uterine inflammation, hemorrhages, leukemia, jaundice, albuminuria, for vaginal hygiene via bathing, or in the form of tincture for topical use directly on skin lesions or even in ointments, creams [10,12], and in the form of soap with an antiacne effect [13]. It is used as an anti-inflammatory, antioxidant, antidiabetic medicine, and a disinfectant [3,10,14,15].

## 4. Chemical Composition

Chemically, *F. chica* is rich in polyphenols, especially flavonoids. Among the flavonoids, derivatives of the class of anthocyanins stand out, which in this plant are abundant in the form of 3-deoxyanthocyanins. These are involved in plant growth and development, including UV protection, stimulation of pollination and seed dispersal, and as a defense mechanism [16]. The 3-deoxyanthocyanins are the substances that give the tea its red color, especially the main compounds carajurin (**1**) and carajurone (**2**) [17], and to a lesser extent four other substances, 3’- hydroxy-carajurone (**3**), 3’-hydroxy-carajurin (**4**), 6,7,3’,4’-tetrahydroxy-5-methoxyflavylium (**5**), and 6,7,4’-trihydroxy-5-methoxyflavylium (**6**) [18]. Substances **1** and **2** were the first to be described in this species.

**Figure 2:**
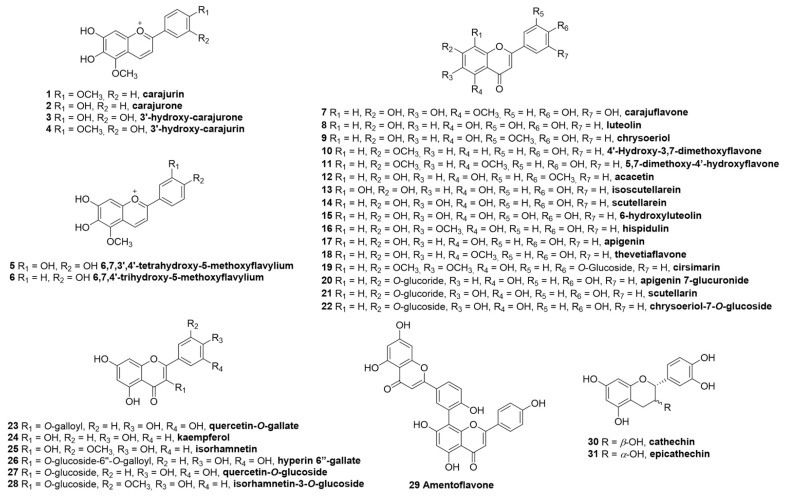
Polyphenols (**1**–**31**) of *F. chica*.

In addition to anthocyanidins, other flavonoids (aglycones and glycosides) have been isolated and/or characterized using high-performance liquid chromatography coupled to mass spectrometry (HPLC-MS) analysis, among them the flavones carajuflavone (**7**), luteolin (**8**) [13], chrysoeriol (**9**) [19], 4′-hydroxy-3,7-dimethoxyflavone (**10**) [20], 5,7-dimethoxy-4′-hydroxyflavone (**11**) [19], acacetin (**12**) [17], isoscutellarein (**13**), scutellarein (**14**), 6-hydroxyluteolin (**15**), hispidulin (**16**), apigenin (**17**) [21], thevetiaflavone (**18**) [13], cirsimarin (**19**) [19], apigenin 7-glucuronide (**20**), scutellarin (**21**) [22], chrysoeriol-*O*-glucoside (**22**) [19]. Flavonols are a more restricted group, being so far characterized as quercetin-*O*-gallate (**23**), kaempferol (**24**), isorhamnetin (**25**) hyperin 6”-gallate (**26**), quercetin-*O*-glucoside (**27**), and isorhamnetin-3-*O*-glucoside (**28**) [19]. The biflavonoid amentoflavone (**29**) and the flavans-3-ols catechin (**30**) and epicatechin (**31**) [19] are other flavonoid derivatives identified in *F. chica* (Figure 2).

**Figure 3:**
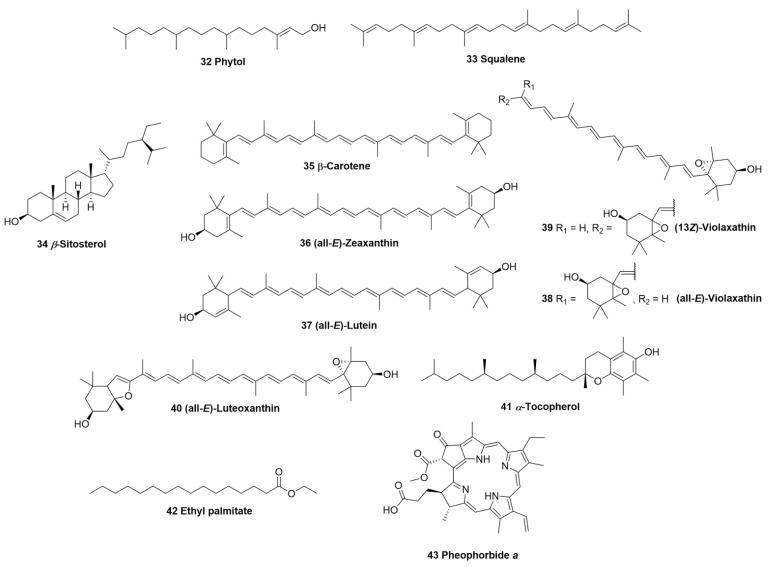
Terpenes (**32**–**40**), tocochromanol (**41**), fatty acid (**42**), and alkaloid (**43**) of *F. chica.*

With respect to other classes of natural products, the characterization of terpenes, including carotenoids, and other classes such as fatty acids and their derivatives, tocochromanols and alkaloids, stands out. The diterpene phytol (**32**), the triterpene squalene (**33**), and the steroidal β-sitosterol (**34**) [23] are described as structures of a terpenic nature of up to 30 carbons described in *F. chica*. On the other hand, a number of tetraterpenes have been identified using HPLC-MS, among them the main compounds β-carotene (**35**), (all-*E*)-zeaxanthin (**36**), (all-*E*)-lutein (**37**), (all-*E*)-violaxathin (**38**), (13*Z*)-violaxathin (**39**), and (all-*E*)-luteoxanthin (**40**) [22]. In addition to antioxidant carotenoids, tocochromanol *α*-tocopherol (**41**) has been described as well [23]. Additionally, the identification of the fatty acid ethyl ester ethyl palmitate (**42**) has been reported [23], as has the alkaloid pheophorbide α (**43**) [24]. In Table 1, the 43 compounds already identified in *F. chica* and their biological activities already studied from these molecules alone (isolated from *F. chica* extract or other sources) are shown. It is worth noting that despite the tea being the most popular use of *F. chica*, phytochemical studies were focused on hydroalcoholic and/or organic extracts, possibly aiming for the enhancement of extraction yields. Moreover, tea preparations may contain the most polar compounds, specifically the positively charged 3-deaoxyanthocyanins, information that is corroborated by the bright red color of the dried leaf infusions. Thus, the composition of the extract is of great importance, as these compounds can exert pharmacological effects with summative, potentiating or deleterious characteristics (Figure 3).

## 5. Pharmacological Properties 

Historically, plants have been an important source of active ingredients for drug development, and scientific research has been directed towards the prospect of new drugs based on natural products [83]. Thus, the connection between plants and health is responsible for the beginning of a new generation of therapy that integrates drugs derived from plants or the use of plants themselves or their parts [84]. The public health system in Brazil offers twelve herbal medicines as treatment options, among them several with anti-inflammatory potential, such as *Aloe vera*, *Schinus terebenthifolius*, and *Uncaria tomentosa* [85,86]. 

The use of plants as a medicine occurs in many traditional societies [87]; therefore, it is important to stimulate scientific studies that prove existing popular knowledge about plants and their effectiveness in the treatment of diseases [88]. In this sense, several studies have been carried out with different extracts of *F. chica* leaves in order to prove the effectiveness of the plant for the therapeutic purposes for which it is popularly used (Table 2).

Among biological effects from *F. chica* leaf extracts, anti-inflammatory, antioxidant, and healing properties are well evidenced from previous studies (Table 2), and result from the leaves’ phytochemical composition. Studies have shown that the compounds identified in *F. chica* leaf extracts, as well as in several other plants, are responsible for the above-mentioned pharmacological activities (Table 1) when evaluated in isolation (Table 1). 

### 5.1. Healing Activity 

The tissue repair process is divided into three successive and overlapping phases that present specific characteristics: (1) the inflammatory phase, (2) the proliferative phase, and (3) the remodeling phase. In the inflammatory phase, there is an increase in vascular permeability, which promotes chemotaxis and the consequent intense migration of leukocytes, mainly neutrophils and macrophages, to the site of tissue damage in response to the release of pro-inflammatory chemical mediators. The proliferative phase is characterized by the reconstitution of epithelial tissue, angiogenesis and migration of endothelial cells, granulation tissue formation, and extracellular matrix deposition, mainly collagen produced by activated fibroblasts. In the remodeling phase, the initial deposited collagen (type III) is degraded by collagenases and reabsorbed, and the gradual replacement of type III collagen by type I collagen occurs until the proportion found in healthy tissue is reached, providing reorganization of the extracellular matrix and marking the success of tissue repair [110,111]. 

Several herbal medicines have been explored in order to evaluate their action on tissue healing, mainly due to their lower cost and fewer observed adverse effects [112]. Tissue regeneration studies with *F. chica* leaves have addressed the action of different extracts and formulations in both in vitro and in vivo experiments using experimental animals in order to investigate different phases of the healing process. Among the main experimental procedures in the bioprospecting of new components with healing potential, cell proliferation assays using fibroblasts and the production of extracellular matrix components such as collagen are considered highly reliable models. Fibroblasts are connective tissue cells that are essential for the formation of the dermis and are responsible for structural firmness. After injury, fibroblasts proliferate and migrate to the injured site, where they produce a large amount of extracellular matrix material, such as type I and III collagen, which helps to isolate and repair the injured tissue. The use of a methanolic extract of *F. chica* leaves stimulates the growth of fibroblasts in vitro in a concentration-dependent manner and increases collagen production [14]. 

From this tissue regeneration property, several in vivo studies with experimental animals have been developed to evaluate the healing potential of *F. chica* in different tissue types. In treatment of gastric ulcers induced by ethanol and indomethacin in Wistar/Uni rats, administration of the hydroalcoholic extract of *F. chica* leaves reduced about 60% of lesions [113]. The methanolic extract administered orally was able to reduce up to 96% of the ulcerative effect of ethanol in a murine model [14]. The ethanol-induced model is associated with gastric mucosal injury induced by oxidative stress (via alcohol); thus, the gastroprotective effect of *F. chica* may occur via antioxidant action. In skin lesions of Swiss mice, treatment with ethanolic extract of *F. chica* leaves has not yet shown promising results. During 21 days of observation, it was found that the extract did not accelerate total tissue regeneration and presented a profile that was similar to the control group [90]. Other studies have investigated the effect of *F. chica* leaves on the healing process of tendon injuries in rats. Observed results included an increase in the total collagen content on the seventh and twenty-first day of the healing process, an increase in the synthesis of collagenase type IV (matrix metalloproteinase-2, an enzyme that participates in the extracellular matrix remodeling process), and better organization of collagen fibers. The treated groups presented a lower inflammatory reaction, which promoted the recovery of the injured animals’ ability to walk [114,115].

In addition to the studies developed in different tissues, investigations of the healing properties of *Fridericia chica* have used different types of formulations for administration of the plant extract and for carrying out these studies. The choice of formulation is extremely important, as it influences the stability, distribution, solubility, bioavailability, and protection of the active compound. Topical formulations such as cream and gel containing *F. chica* leaf extract are being developed for the treatment of skin lesions [90,116]. For the treatment of gastric ulcers, a nanoformulation of *F. chica* extract has been elaborated in chitosan triphosphate-based nanoparticles for oral administration. Nanoformulation was responsible for a greater fibroblast proliferation effect, and the anti-ulcerogenic effect of *F. chica* leaf extract in a nanoformulation was more efficient compared to hydroalcoholic extract [113].

From the 43 compounds identified from *F. chica* leaves, seven molecules (comprising the classes of flavones, flavonols, terpenes, tochromanols, and alkaloids) have been described to present healing properties when evaluated alone. Among them, kaempferol (compound **24**—Table 1) is a flavonol which has been described as improving repair of diabetic excisional and nondiabetic incisional wounds in rats, working as an effective topical wound healing agent [52]. Another component present in *F. chica* leaf extract in considering amount is α-tocopherol (compound **41**, Table 1), belonging to the tocochromanol class. Shuid and colleagues [78] evaluated the effects of *α*-tocopherol supplementation on osteoporotic fracture healing in a rat model, showing an improvement in fracture healing associated with the antioxidatant property. Other compounds have been shown to induce healing activity, such as apigenin (compound **17**, Table 1), with anti-hyaluronidase and anti-collagenase activities [41], scutellarin (compound **21**, Table 1) with angiogenic properties [49], and pheophorbide *a* (compound **43**, Table 1), which decreased the length of the inflammation stage [82].

### 5.2. Anti-Inflammatory Activity 

Inflammation is defined as a protective response of the host to various interactions with the external environment. The assembly of this response requires the participation of cells and molecules, including macrophages and neutrophils, along with cytokines, such as interleukin (IL)-1β and IL-6 [117,118,119,120,121]. These cells recognize and discriminate stimuli as damage-associated molecular patterns (DAMPs) and pathogen-associated molecular patterns (PAMPs) through pattern recognition receptors (PRR) [122]. The inflammatory response, despite being an effective defense mechanism, can occur in an exacerbated or perpetuated manner for a longer period of time than is necessary, causing important tissue damage and contributing to the development of diseases such as rheumatoid arthritis [123,124].

The participation of inflammation in the pathophysiology of multiple diseases stimulates the constant development of anti-inflammatory drugs, and nature represents an important source of plants with therapeutic potential that needs to be explored [125,126]. Plants such as *Aloe vera* have demonstrated efficacy in controlling the inflammatory response and are considered by the Brazilian public health system as a treatment option [127]. Flavonoids, a class of secondary metabolites belonging to the group of phenolic compounds, are anti-inflammatory agents and can act to block arachidonic acid cascade through inhibition of the COX and lipoxygenase pathways [128,129]. The species *Fridericia chica* is used in traditional medicine as an anti-inflammatory agent, which has been confirmed in several studies using different extracts prepared from the leaves of this plant [15,17,130].

The chemical characterization of extracts of *F. chica* leaves has allowed the identification of 3-deoxyanthocyanidins, to which are attributed the greater part of the anti-inflammatory effect of this plant [4,17,18]. In 2001, Zorn et al. [17] demonstrated that both the lipophilic extract of *Fridericia chica* leaves (200 mg/mL) and one of its isolated components (carajurine) at a concentration of 500 µM had the ability to completely inhibit nuclear factor kappa B (NF-κB), which is responsible for the transcription of genes that encode several pro-inflammatory mediators and which participates in the pathogenesis of diseases such as multiple sclerosis [131,132,133]. Several authors have suggested that other unidentified substances present in the extract of *F. chica* leaves may contribute to the anti-inflammatory activity of this species [17].

Several models of inflammation have been used for the study of anti-inflammatory drugs, one of which is the experimental model of inflammation induced by snake venom. Its pathogenesis may involve the participation of the enzyme phospholipase A_2_, which stimulates the release of arachidonic acid [134,135]. In 2009, Oliveira et al. [15] evaluated the anti-inflammatory effect of an aqueous extract of the leaves of *Fridericia chica* in mice inoculated with the venom of the snakes *Bothrops atrox* and *Crotalus durissus ruruima*, administered via the subcutaneous and intraperitoneal routes at different times. After treatment with the extract, it was possible to observe that it showed inhibitory activity, especially in the processes of myocytolysis and granulocyte migration, thus resulting in a decrease in inflammation. This effect is attributed to substances present in the extract, flavonoids among them.

The effectiveness of *Fridericia chica* leaves as an anti-inflammatory agent has been demonstrated in other models, such as inflammatory angiogenesis, which was induced in a murine model by a sponge implant (polyether-polyurethane) and in the proliferative process of human tumor cell lines in vitro, in which Michel et al. (2015) [130] observed a reduction in the amount of neutrophils at the site of the sponge implants and a great decrease in angiogenesis after treatment with aqueous and ethanolic extracts. Flavone escutelarin derivatives, found in the hydroethanolic extract of *Fridericia chica* leaves, have been associated with a reduction of parameters linked to inflammatory response in the model of intestinal mucositis induced by 5-fluorouracil in a study by [136].

Among phytochemical compounds present in *F. chica* leaf extracts, 25 have been described as presenting anti-inflammatory behavior (Table 1). The anthocyanins comprise the class of most abundant compounds in *F. chica* leaves, with carajurin composing one of the major molecules found in extract of the leaves [17]. Carajurin (compound 1, Table 1) is capable of inhibiting transcription factor NF-κB, an important signaling pathway associated with pro-inflammatory response [17]. *F. chica* leaf extracts consist of a high range of flavonoid compounds, such as flavones and flavonols (Table 1). Regarding this class, the literature on their biological potentials is extensive, with a total of 14 flavonoids from *F. chica* leaf extracts having been shown to suppress inflammation through different cell signaling pathways. An example is luteolin (compound **8**, Table 1), a flavone which has been shown to interact with several inflammatory targets such as matrix metalloprotease 9 (MMP9) and mitogen-activated protein kinase 1 (MAPK1) [25]. Chrysoeriol (compound **9**, Table 1), another flavone, ameliorates TPA-induced ear edema in mice, and its inhibition of JAK2/STAT3 and IκB/p65 NF-κB pathways is involved in the anti-inflammatory effects [27]. Hispidulin, a flavone found in a number of plants, among them in *F. chica*, was evaluated by Yu et al. (2020) [36] in relation to protection against neuroinflammation using the immortalized cell line of murine microglial BV2 (BV2 cells). Hispidulin has been shown to inhibit NO and ROS production, which suppressed the expression of the inflammation-related enzymes iNOS and COX-2 [137] in a dose-dependent manner. Corroborating these data, Torres et al. (2018) [138] showed a decrease in the enzymatic activity of lipoxygenase in the presence of ethanolic extract of *F. chica* leaves. They observed that treatment with hispidulin in the presence of LPS inhibited the production of TNF- α, IL-6, IL-1 β, and PGE2 in a dose-dependent manner. In addition, hispidulin showed activity in reducing NF-κB, and decreased the levels of phosphorylation of IκB kinase in BV2 cells. Based on the above examples, there is other evidence of the effect of the species *F. chica* leaves in the modulation of the cytokine profile, among them that demonstrated by Lima (2020) [139], which suggests an increase in the production of IL-10 and a decrease in the production of IL-1β in RAW 264.7 cells activated with LPS in the presence of a hydroethanolic extract of *F. chica*. The authors attributed this effect to 5 *O*-methylescutelarin, a derivative of the flavone escutelarin. The aforementioned studies corroborate the therapeutic potential described in traditional medicine for *F. chica*.

Despite being encountered in minor proportions in *F. chica*, terpenes may play roles in its biological activities, including in anti-inflammatory response. β-carotene (compound **35**, Table 1) was capable of inhibiting LPS-stimulated Cox2, Nos2, and Tnfalpha gene expression from macrophages in vitro [69]. Another terpene, phytol (compound **32**, Table 1), has been described as inducing anti-inflammatory activity in vitro and in vivo, with higher potency in the presence of others non-steroidal anti-inflammatories [64]. Among fatty acids, ethyl palmitate (compound **42**, Table 1) reduced plasma levels of tumor necrosis factor-α (TNF-α) and IL-6, decreased NF-κB expression in liver and lung tissues, and ameliorated histopathological changes in an LPS-induced endotoxemia rat model [79]. The alkaloid pheophorbide *a* (compound **43**, Table 1), was found to inhibit nitric oxide production and suppress the expression of iNOS proteins in LPS-stimulated macrophages [80].

### 5.3. Antioxidant Activity

The term oxidative stress, first proposed in 1985, refers to the imbalance between the production of oxidizing and antioxidant molecules; it favors oxidation reactions and compromises cell signaling and redox control [140,141,142]. The aforementioned changes result in excessive production of free radicals, which can contribute to the generation of reactive oxygen species, thus resulting in structural damage to biological systems [143,144,145]. Reactive species are defined as atoms, molecules, or ions resulting from oxygen, usually with a high reactivity capacity, which can cause damage to cellular constituents, such as lipid peroxidation and oxidation of DNA bases [146,147,148,149]. Free radicals and other reactive species are essential for cellular homeostasis, but when generated excessively they contribute to cellular aging and the development of diseases, as is observed in the development of hemolytic anemia due to deficiency of the enzyme glucose-6-phosphate dehydrogenase. In addition, oxidative stress is part of the pathophysiology of several chronic degenerative diseases, including cancer, diabetes, and hypertension [150,151,152,153,154,155,156].

Antioxidants are compounds that are capable of slowing, preventing, or removing damage caused by redox imbalance, and thereby regulating the development of oxidative stress [157]. The large number of adverse effects observed with the use of synthetic antioxidants have driven the search for other alternatives, among them substances isolated from natural products [158,159,160]. The main antioxidants present in plants are phenolic compounds, which represent a family of secondary metabolites with the property of interrupting chain reactions caused by free radicals by donating hydrogen atoms or electrons [161,162,163]. To this large group of compounds belong several of the flavonoids, which are present in medicinal plants such as the species *F. chica* [20,21,91,164,165,166].

The antioxidant potential of *F. chica* has already been evaluated in several studies. In 2012, Do Amaral et al. [109] identified the presence of phenolic compounds in an ethanolic extract of the leaves of this plant. In addition, Gemelli et al. (2015) [166] and Campos de Siqueira et al. (2019) [22] examined the antioxidant potential in the aqueous and hydroethanolic leaf extract, respectively. The anti-inflammatory property of 3-deoxyanthocyanidins, which are phenolic pigments with the ability to stabilize free radicals by donating hydrogen radicals, was proposed by Zorn et al. in 2001 [17]. However, the antioxidant capacity of these flavonoids was identified much later, by Do Amaral et al. in 2012 [109], and later confirmed by Dos Santos et al. in 2013 [166]. Another important finding was that reported by Olivero-Verbel et al. in 2021 [167], which suggests the ability of aqueous extract of *F. chica* leaves to induce nuclear translocation of DAF-16 in a prominent manner, thus suggesting the potential of this extract in the regulation of oxidative stress [17,22,109,166,167,168].

As already mentioned, flavonoids are phenolic compounds which have a great antioxidant potential. Among them are flavones, the antioxidant capacity of which is related to the presence of free hydroxyl groups in their A and B rings [161,162]. Luteolin (compound **8**—Table 1), one of the most abundant flavones, was identified by Do Amaral et al. (2012) [116], who evaluated the antioxidant activity of the ethanolic extract of *F. chica* leaves in the presence of the free radical DPPH (2,2-diphenyl-1-picrylhydrazil) using *Gingkgo bibola* as a positive control and demonstrated that the antioxidant capacity of *F. chica* extract is superior. This antioxidant effect was attributed mainly to the flavones luteolin and apigenin. In 2015, Gemelli et al. [168] confirmed the presence of luteolin in the aqueous extract of *F. chica* leaves and demonstrated its antioxidant potential by DNA damage assay in CHO cells (hamster ovary cells) [109,168].

The leaves of *Fridericia chica* have a high flavones content along with the presence of apigenin (compound **17**, Table 1), with high antioxidant activity [40], as evidenced by Siraichi et al. in 2013 [21], confirming what had already been proposed by Do Amaral et al. in 2012 [109]. Other important flavone identified in the extract of *F. chica* leaves are scutellarin (compound **21**, Table 1) and scutellarein (compound **14**, Table 1), which are present in other medicinal plants such as *Scutellaria barbata* and *S. lateriflora*. Scutellarin is a glucoronid form of scutellarein, which means that although both molecules present antioxidant activity, the aglycone form (scutellarein) has a stronger activity [34]. The research of Campos de Siqueira et al. (2019) [22], who identified carotenoids such as β-carotene and α-carotene in a hydrometanolic extract of *F. chica*, was not the first time that escutelarin was identified in the species *F. chica*, as in 2013 Siraichi et al. [21] attributed the antioxidant effect of the species to the presence of this flavone [21,22,109].

Terpenes are well known antioxidant agents, and have been shown to provide relevant protection under oxidative stress conditions. The terpene zeaxanthin (compound **36**, Table 1) protects against chronic eye and cardiovascular diseases due its antioxidant property by directly quenching reactive oxygen species (ROS) and by facilitating glutathione synthesis [72]. Violaxanthin (compound **38**, Table 1) possesses potent lipid peroxidation inhibitory activity [76]. Other compounds from different classes, such as *α*-Tocopherol and Pheophorbide *a* (compounds **41** and **43**, respectively, in Table 1) have shown antioxidant activity as well, and are present in others plant species [77,81].

Martins et al., in 2016 [169] and Ribeiro et al., working in 2018 [170], demonstrated the antioxidant capacity of a hydroethanolic extract of *Fridericia chica* in the presence of both the free radical DPPH and the oxidative damage induced by ultraviolet radiation. The use of several methodologies to evaluate the effects of *F. chica* extracts for the control of oxidative stress allowed for a more complete view of this mechanism. Other authors, such as Torres et al. in 2018 [138] and Teixeira et al. in 2017 [171], have included other free radical neutralization assays, such as ABTS (2,2’-Azino-bis (3-ethylbenzothiazoline-6-sulfonic acid) and FRAP (ferric reducing antioxidant power), thus reaffirming the biological effects of *F. chica* [138,169,170,171].

In 2009, the Brazilian Ministry of Health published a list of 71 species that are part of the Report on Medicinal Plants of Interest to the SUS (RENISUS); *F. chica* is among them due to the large amount of scientific evidence pointing to its different biological effects [172,173]. One of the most important biological effects of this species is the antioxidant effect, which can contribute to the control and prevention of degenerative diseases, as already mentioned. Encouraging the development of research related to *F. chica* is be essential for its inclusion in the list of herbal medicines prescribed within the Brazilian health services, through which twelve herbal medicines are already available that are part of the National List of Essential Medicines (RENAME) [127].

## 6. Conclusions

The World Health Organization (WHO) has encouraged the inclusion of medicinal plants in primary health care networks, and in Brazil their use is regulated by the National Health Surveillance Agency (ANVISA). The public health system of Brazil has offered herbal medicines since 2007, and 12 herbal medicines have been made available in the public network by the Ministry of Health (MS). The species *Fridericia chica* (Bonpl.) L. G. Lohmann is traditionally used in folk medicine, especially in the Amazon region, and is part of the 71 species of interest to the SUS (Brazilian public health system). Among these species, *Aloe vera*, *Schinus terebenthifolius*, and *Uncaria tomentosa* are used as commercial herbal pharmaceutical formulations in the Brazilian Health System. *Schinus terebenthifolius* has healing and anti-inflammatory action and is used as an antiseptic topical for gynecological applications, available in the forms of cream and ovules. *Uncaria tomentosa* is indicated as an assistant in cases of arthritis and osteoarthritis thanks its anti-inflammatory and immunomodulatory activity, and is available in capsule, pill, and gel forms. *Aloe vera* is indicated for the topical treatment of first and second degree burns and as an adjunct in cases of psoriasis vulgaris, and is available in cream form [85]. Therefore, the present review indicates the potential inclusion of *F. chica* on the list of herbal medicines prescribed within the scope of Brazilian Health System services, as the extract can be used in different pharmacological formulations such as topical or oral administrations in order to treat diseases associated with inflammatory response and wounded tissue.

Scientific evidence indicates the different biological effects of *F. chica*, which proves the therapeutic effect of this species in folk medicine, which is explained especially by the presence of a wide range of biologically active compounds that are known from other plant species. The present review has shown that from all compounds identified in *F. chica* leaf extract, over 70% are known to exert anti-inflammatory, antioxidant, and/or healing properties based on previous studies. Therefore, the pharmacological properties of *F. chica* leaf extract are directly associated with the phytochemical composition, with different molecules working in association in order to observe the conclusive therapeutical potential. Although carajurin is found as the major component of the extract with anti-inflammatory activity, its synergism with other less abundant components is important to the overall pharmacological properties of the extract. Therefore, the proportion of every molecule within the extract and its biological potency should be more closely investigated. It is of great importance to encourage more studies related to the chemical profile, standardization of extracts, and proof of pharmacological activities of *F. chica*. Concerning the traditional use of *F. chica* leaf extract as tea, more studies should be performed in order to evaluate the phytochemical composition of aqueous infusions, considering that almost all studies on leaves have used extract compositions with other types of solvents. New research needs to be developed in order to add scientific evidence that favors the use of this and other medicinal species, both in Brazil and in other regions of the world.

The scientific investigations cited in this study contribute to the construction of arguments, providing a solid basis to attribute anti-inflammatory, antioxidant, and healing properties to *Fridericia chica*, thus demonstrating the scientific bases that justify its use in traditional medicine.

## Figures and Tables

**Table 1:** Phytochemical components of *F. chica* extracts and their biological activities.

Compound No.	Name	Source	Isolation/Detection	Biological Activity *
*Anthocyanins*				
**1**	Carajurin	leaves, MeOH	Isolation [17]	Anti-inflammatory [17]
**2**	Carajurone	leaves, MeOH	Isolation [17]	N.A.
**3**	3’-Hydroxy-carajurone	leaves, MeOH	Isolation [17]	N.A.
**4**	3’-Hydroxy-carajurin	leaves, MeOH	Isolation [17]	N.A.
** 5**	6,7,3’,4’-Tetrahydroxy-5-methoxyflavylium	leaves, DCM fraction	Isolation [18]	N.A.
**6**	6,7,4’-Trihydroxy-5-methoxyflavylium	leaves, DCM fraction	Isolation [18]	N.A.
*Flavones*				
**7**	Carajuflavone	leaves, AcOEt fraction	Isolation [1]	N.A.
**8**	Luteolin	leaves, AcOEt fraction	Isolation [1]	Anti-inflammatory [25] Antioxidant [26]
** 9**	Chrysoeriol	leaves, 70% EtOH	HPLC-MS detection [19]	Anti-inflammatory [27] Antioxidant [28]
**10**	4′-Hydroxy-3,7-dimethoxyflavone	leaves, EtOH	Isolation [20]	N.A.
**11**	5,7-dimethoxy-4′-hydroxyflavone	leaves, 70% EtOH	HPLC-MS detection [19]	N.A.
**12**	Acacetin	leaves, MeOH	Isolation [17]	Anti-inflammatory [29] Antioxidant [30]
**13**	Isoscutellarein	leaves, 90% EtOH	HPLC-MS detection [21]	Antioxidant [31]
**14**	Scutellarein	leaves, 90% EtOH	HPLC-MS detection, isolation [21]	Anti-inflammatory [32,33] Antioxidant [34]
**15**	6-Hydroxyluteolin	leaves, 90% EtOH	HPLC-MS detection [21]	N.A.
** 16**	Hispidulin	leaves, 90% EtOH	HPLC-MS detection [21]	Anti-inflammatory [35,36] Antioxidant [37]
**17**	Apigenin	leaves, 90% EtOH	HPLC-MS detection, isolation [21]	Anti-inflammatory [38,39] Antioxidant [40] Healing [41]
**18**	Thevetiaflavone	leaves, AcOEt fraction	Isolation [1]	Antioxidant [42]
**19**	Cirsimarin	leaves, 70% EtOH	HPLC-MS detection [19]	Anti-inflammatory [43] Antioxidant [44] Healing [45]
**20**	Apigenin 7-glucuronide	leaves, 80% MeOH	HPLC-MS detection [22]	Anti-inflammatory [46] Antioxidant [47]
**21**	Scutellarin	leaves, 80% MeOH	HPLC-MS detection [22]	Anti-inflammatory [48] Antioxidant [34] Healing [49]
**22**	Chrysoeriol-*O*-glucoside	leaves, 70% EtOH	HPLC-MS detection [19]	N.A.
*Flavonols*				
**23**	Quercetin-*O*-gallate	leaves, 70% EtOH	HPLC-MS detection [19]	N.A.
**24**	Kaempferol	leaves, 70% EtOH	HPLC-MS detection [19]	Anti-inflammatory [50,51] Antioxidant [31] Healing [52]
**25**	Isorhamnetin	leaves, 70% EtOH	HPLC-MS detection [19]	Anti-inflammatory [53] Antioxidant [54]
**26**	Hyperin 6”-gallate	leaves, 70% EtOH	HPLC-MS detection [19]	Antioxidant [55]
**27**	Quercetin-*O*-glucoside	leaves, 70% EtOH	HPLC-MS detection [19]	Antioxidant [56]
**28**	Isorhamnetin-3-*O*-glucoside	leaves, 70% EtOH	HPLC-MS detection [19]	Antioxidant [57]
*Flavone dimer*				
**29**	Amentoflavone	leaves, 70% EtOH	HPLC-MS detection [19]	Anti-inflammatory [58] Antioxidant [59]
*Flavan-3-ols*				
**30**	Catechin	leaves, 70% EtOH	HPLC-MS detection [19]	Anti-inflammatory [60] Anti-oxidant [61]
**31**	Epicatechin	leaves, 70% EtOH	HPLC-MS detection [19]	Anti-inflammatory [62] Antioxidant [63]
*Terpenes*				
**32**	Phytol	leaves, 70% EtOH, hexane fraction	GC-MS detection [23]	Anti-inflammatory [64] Antioxidant [65]
**33**	Squalene	leaves, 70% EtOH, hexane fraction	GC-MS detection [23]	Antioxidant [66] Anti-inflammatory [67]
**34**	β-Sitosterol	leaves, 70% EtOH, hexane fraction	GC-MS detection [23]	Anti-inflammatory [68]
**35**	β-Carotene	leaves, acetone	HPLC-MS detection [22]	Anti-inflammatory [69] Antioxidant [69] Healing [70]
**36**	(all-*E*)-Zeaxanthin	leaves, acetone	HPLC-MS detection [22]	Anti-inflammatory [71] Antioxidant [72]
**37**	(all-*E*)-Lutein	leaves, acetone	HPLC-MS detection [22]	Anti-inflammatory [73] Antioxidant [74]
**38**	(all-*E*)-Violaxanthin	leaves, acetone	HPLC-MS detection [22]	Anti-inflammatory [75] Antioxidant [75]
**39**	(13*Z*)-Violaxanthin	leaves, acetone	HPLC-MS detection [22]	Antioxidant [76]
**40**	(all-*E*)-Luteoxanthin	leaves, acetone	HPLC-MS detection [22]	N.A.
*Tocochromanol*				
**41**	*α*-Tocopherol	leaves, 70% EtOH, hexane fraction	GC-MS detection [23]	Antioxidant [77] Healing [78]
*Fatty acid*				
**42**	Ethyl palmitate	leaves, 70% EtOH, hexane fraction	GC-MS detection [23]	Anti-inflammatory [79]
*Alkaloid*				
**43**	Pheophorbide *a*	leaves, 90% EtOH	Isolation [24]	Anti-inflammatory [80] Antioxidant [81] Healing [82]

* Biological activities concern anti-inflammatory, antioxidant, and healing activity described in previous articles using the isolated components obtained from *F. chica* or other plants. N.A., not available.

**Table 2:** Pharmacological properties of extracts of *Fridericia chica*.

Authors	Type of Study	Action	Etiological Agent	Plant Material/Part	Treatment
[20]	In vitro study	Antifungal and antiprotozoal	Trichophyton mentagrophytes (fungus) and *Trypanosoma cruz*i	Ethanolic extract of leaves	4 mg/mL (trypanocide); 3.125 mg/mL (fungicide)
[89]	In vitro study	Antiprotozoal	*Leishmania amazonensis* and *Leishmania infantum*	Hexanic leaf extract	37.2 µg/mL (*L. amazonensis*); 18.6 µg/mL (*L. infantum*)
[90]	In vitro and in vivo study	Antiprotozoal and healing	*Leishmania amazonensi*; Swiss Webster mouse	Ethanolic extract of leaves and fractions	Leishmanicidal effect: 60–155.9 μg/mL; healing effect: 10 mg/g
[24]	In vitro study	Antiprotozoal	*Trypanosoma cruzi*	Hydroethanolic extract of leaves and fractions	24.8 μg/mL–213 μg/mL (hydroethanolic extract); 2.3 μg/mL–10 μg/mL (feoforbide-a)
[91]	In vivo Study	Antimicrobial	*Helicobacter pylori* 43,504 and *Enterococcus faecalis* 29,212	Hydroethanolic extract of leaves	12.5 μg/mL (*H. pylori*); 100 μg/mL (*E. faecalis*)
[92]	In vitro study	Antimicrobial	*Staphylococcus* sp.	Hydroalcoholic extract	250 μg gallic acid equivalent (GAE)/mL–MIC; 1000 μg GAE/mL–MBC
[93]	In vitro study	Antimicrobial	*Candida* sp.	Dichloromethane extract of leaves	0.007–0.03 mg/mL
[94]	In vitro study	Antiviral	aMPV cepa SHS/669/03	Ethanolic extract of leaves	2.5 μg/mL
[95]	In vitro study	Antiviral	Human Herpes Virus type 1 (HHV-1); murine Encephalomyocarditis virus (EMCV); Vaccinia Virus strain Western Reserve (VACV-WR)	Ethanolic extract of leaves	EC_50_: 245.7 μg/mL (HHV-1); 86.3 μg/mL (VACV-WR)
[96]	In vivo study	Antitumor	Solid Ehrlich tumor	Ethanolic extract and aqueous extract of leaves	30 mg/kg body weight (10 days of oral treatment)
[97]	In vivo study	Antitumor	7,12-dimethyl injection-induced breast cancer-1,2-benzanthracene (DMBA)	Hydroalcoholic extract of leaves	Oral administration for 16 weeks: extract at a dose of 300 mg/kg; 7,12-dimethyl-1,2-benzanthracene (DMBA) associated with vincristine 250 µg /mL
[98]	In vitro study	Antitumor and fibroblast proliferation	Human tumor cell lines: MCF-7 (breast), NCI-ADR/RES (ovary with multiple drug resistance phenotype), UACC-62 (melanoma), NCI–h460 (lung), PC-3 (prostate), HT29 (colon), OVCAR-03 (ovary), 786-0 (kidney) and K562 (leukemia). Fibroblasts: obtained from 3T3 mice	Crude leaf extracts (without and with enzyme treatment)	0.25; 2.5 and 25 μg/mL without enzymatic treatment (fibroblast proliferation); 7.4 and 8.7 μg/mL with enzymatic treatment (cytostatic effect for UACC-62–melanoma lineage)
[99]	Survey of use by health professionals	Anti-inflammatory	Oral diseases	Leaves	Tea (no dose determined)
[19]	In vivo study	Analgesic and anti-inflammatory	Osteoarthritis induced by sodium monoiodoacetate	Hydroalcoholic extract of leaves	Oral administration s. i.d. for 25 days: 50 mg/kg, 150 mg/kg, 450 mg/kg
[100]	Survey of studies with herbal medicines	Anti-acne	Does not apply	Not mentioned	Not informed
[101]	Survey of traditional use	Treatment of skin irritation and healing	Measles and smallpox	Leaves	Infusion and bath (measles and smallpox); leaves macerated and applied to the affected area (lesions)
[102]	Review	Treatment of tuberculosis-related symptoms	Mycobacterium tuberculosis	Not specified	Not specified
[103]	Survey of traditional use	Anemia and weakness	Does not apply	Leaves	Not specified
[104]	In vivo study	Antihypertensive	Does not apply	Hydroalcoholic extract of leaves	Oral administration of the extract at doses of 100 mg/kg; 250 mg/ kg and 500 mg/kg
[105]	Survey of traditional use	(1) Anemia, weakness, restoration facial color in malaria patients; (2) ovarian cysts, cystitis, hepatitis, liver, diarrhea; (3) flu, cough, anemia; (4) aids getting pregnant, ulcers, (5) vaginal itching	Does not apply	Leaves	(1) maceration or tea; (2) tea or infusion; (3) syrup; (4) bottled; (5) bath. No dose determined
[21]	Ex vivo study	Photoprotection	Does not apply	Various parts	Topical application of nonionic cream with 2.5% ethylacetate fraction and 2.5% hexane fraction
[106]	In vitro study	Photosensitization	MCF-7 cells of human breast adenocarcinoma	Extract nanoemulsion produced from aerial parts	CC_50_: 1.3 μg ACE/mL
[107]	In vitro study	Anti-hepatoxic	Does not apply	Leaves	0.25–1.25 mg/mL
[108]	In vivo study	Anti-hepatoxic	Carbon tetrachloride	Ethanolic extract of leaves	300, 500 and 600 mg/kg
[109]	In vitro study	Antioxidant	Free Radical DPPH (1,1-diphenyl-2-picrylidazyl	Ethanolic extract of leaves and fractions	5, 10, 25, 50, 125 and 250 µg/mLin ethanol
[14]	In vitro study	Antioxidant	Free Radical DPPH (1,1-diphenyl-2-picrylidazyl	Methanolic extract of leaves	0.25; 2.5; 25 and 250 μg/mL

## Data Availability

Not applicable.
